# The Health of Louisville

**Published:** 1851-11

**Authors:** 


### THE HEALTH OF LOUISVILLE.

At no time during our residence in Louisville, have we ever known the sanitary condition of the city more perfect than at present. There is scarcely any disease in the city, except fever and ague, and we have never known less of that disease, at this season of the year. The sparse attacks of that autumnal visitant are confined, almost entirely, to portions of the suburbs. B.

